# Roles of ubiquitin-specific proteases in inflammatory diseases

**DOI:** 10.3389/fimmu.2024.1258740

**Published:** 2024-01-23

**Authors:** Rui Chen, Hui Zhang, Linke Li, Jinsheng Li, Jiang Xie, Jie Weng, Huan Tan, Yanjun Liu, Tailin Guo, Mengyuan Wang

**Affiliations:** ^1^ Center of Obesity and Metabolic Diseases, Department of General Surgery, The Third People's Hospital of Chengdu, Affiliated Hospital of Southwest Jiaotong University, Chengdu, China; ^2^ Department of Stomatology, The Third People's Hospital of Chengdu, The Affiliated Hospital of Southwest Jiaotong University, Chengdu, Sichuan, China; ^3^ College of Medicine, Southwest Jiaotong University, Chengdu, Sichuan, China; ^4^ College of Materials Science and Engineering, Southwest Jiaotong University, Chengdu, Sichuan, China; ^5^ Department of Pediatrics, Chengdu Third People's Hospital, Chengdu, Sichuan, China

**Keywords:** deubiquitination, ubiquitin-specific proteases, inflammatory diseases, protein stability, targeted therapies

## Abstract

Ubiquitin-specific proteases (USPs), as one of the deubiquitinating enzymes (DUBs) families, regulate the fate of proteins and signaling pathway transduction by removing ubiquitin chains from the target proteins. USPs are essential for the modulation of a variety of physiological processes, such as DNA repair, cell metabolism and differentiation, epigenetic modulations as well as protein stability. Recently, extensive research has demonstrated that USPs exert a significant impact on innate and adaptive immune reactions, metabolic syndromes, inflammatory disorders, and infection via post-translational modification processes. This review summarizes the important roles of the USPs in the onset and progression of inflammatory diseases, including periodontitis, pneumonia, atherosclerosis, inflammatory bowel disease, sepsis, hepatitis, diabetes, and obesity. Moreover, we highlight a comprehensive overview of the pathogenesis of USPs in these inflammatory diseases as well as post-translational modifications in the inflammatory responses and pave the way for future prospect of targeted therapies in these inflammatory diseases.

## 1 Introduction

**Figure 1:**
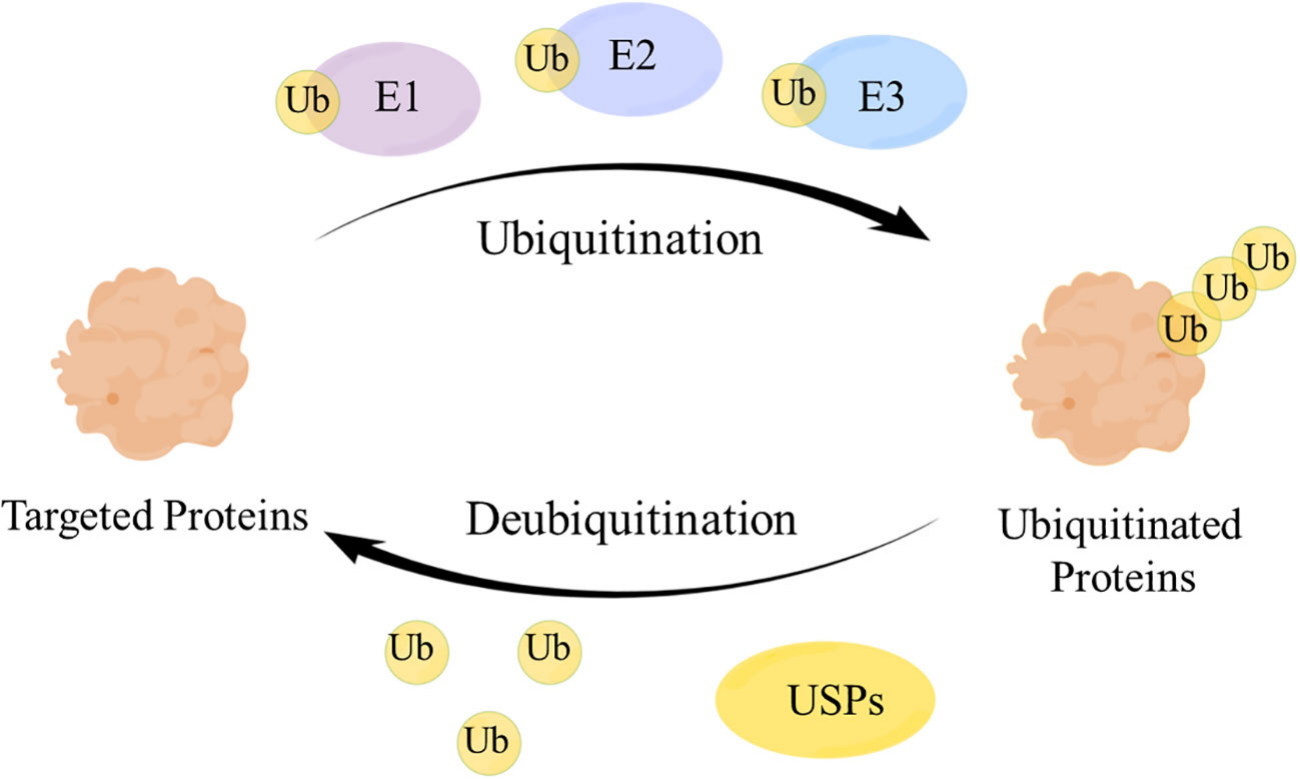
The mechanism of the ubiquitin-protease system. Ubiquitin (Ub) attaches to the targeted proteins by E1, E2, E3 enzymes to realize the ubiquitination of targeted proteins. Ubiquitin-specific proteases (USPs) remove Ub from the ubiquitinated proteins to regulate the fate of proteins and signaling pathway transduction.

Deubiquitinating enzymes (DUBs), a large group of the ubiquitin system (1), have ubiquitin binding motifs (2), which ensure specific and rigorous regulation by assisting the recognition and recruitment of ubiquitinated proteins (3). Ubiquitin (Ub) attaches to proteins by cascades of E1 (activating), E2 (conjugating), and E3 (ligating) enzymes and regulates protein interactions (4), while DUBs remove Ub from ubiquitinated proteins through splitting the peptide or isopeptide bonds between Ub and its substrates, thereby stabilizing the substrate proteins and reversing ubiquitination (5) (**Figure 1**). DUBs can avoid over-activation of the signaling pathway by modulating the signal transduction and have a significant impact on maintaining the balance of the ubiquitin system (6).

**Figure 2:**
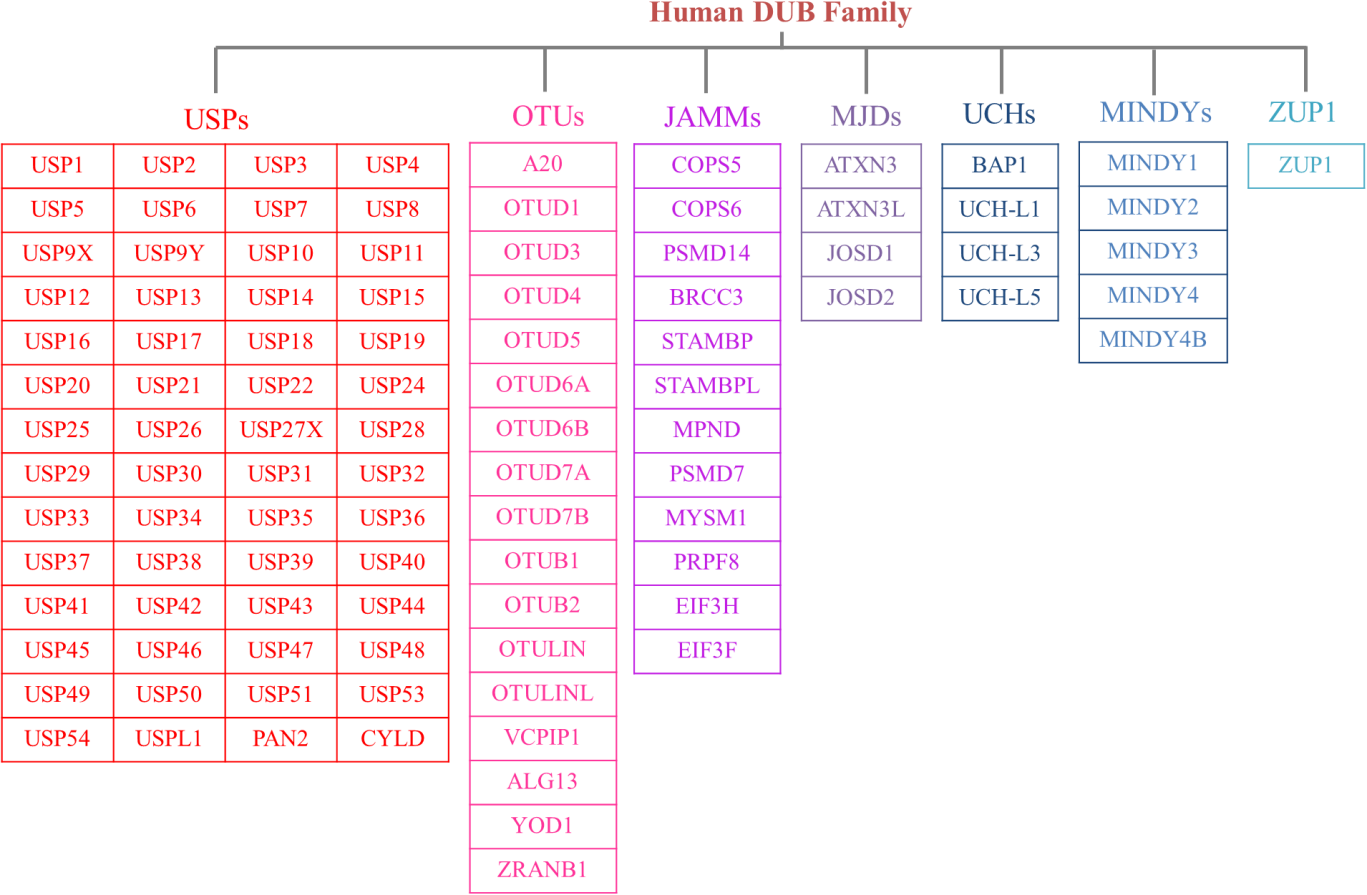
The members of DUB family. According to sequence and structural similarity, DUBs are classified into seven families, including the Ubiquitin-specific proteases (USPs), Ovarian tumor proteases (OTUs), Josephins and JAB1/MPN/Mov34 metalloenzymes (JAMMs), Machado-Josephin disease proteins (MJDs), Ubiquitin carboxyl-terminal hydrolases (UCHs), motif interacting with ubiquitin-containing novel DUB family proteases (MINDYs) and zinc finger-containing ubiquitin peptidase 1 (ZUP1). And the USP family is the largest group of the DUBs.

The human genome contains nearly 100 DUBs. They are classified into seven families based on their sequence and structural similarity, including the Ubiquitin-specific proteases (USPs), Ovarian tumor proteases (OTUs), Josephins and JAB1/MPN/Mov34 metalloenzymes (JAMMs), Machado-Josephin disease proteins (MJDs), Ubiquitin carboxyl-terminal hydrolases (UCHs), motif interacting with ubiquitin-containing novel DUB family proteases (MINDYs) and zinc finger-containing ubiquitin peptidase 1 (ZUP1) (6, 7) (**Figure 2**). Among these families, USPs have the largest number of members and play crucial roles in regulating protein fate and signaling pathway transduction by removing Ub from targeted proteins (8). Furthermore, USPs are essential for the modulation of a variety of physiological processes, such as DNA repair, cell metabolism and differentiation, epigenetic modulations as well as protein stability (9, 10). Recent studies have highlighted the significant impact of USPs on innate and adaptive immune reactions, metabolic syndromes, inflammatory disorders, and infections through post-translational modification (11). Dysregulation of USPs has been observed in several inflammatory diseases, suggesting their potential involvement in the underlying mechanisms (12).

Inflammation, which is triggered by various molecules and signaling pathways, serves as a defense response of the immune system to pathogen infection and tissue damage (13). Inflammation is like a double-edged sword. Moderate inflammatory reaction can protect the human body, while excessive inflammatory response will be harmful (14). Numerous inflammatory diseases, such as periodontitis, pneumonia, inflammatory bowel disease, and hepatitis, pose significant challenges to maintaining tissue homeostasis and preventing damage (15). Traditional treatments for inflammation often involve extensive immune suppression or direct cell eradication, which can compromise the immune system and increase the risk of infections (13). The roles of USPs in inflammatory diseases have garnered increasing attention, with extensive research suggesting their involvement in the onset and progression of inflammatory responses (12). However, due to the diverse functions and importance of USPs, further exploration is required to fully understand their precise roles in inflammatory diseases (5). Therefore, gaining a comprehensive understanding of the contribution of USPs to inflammatory diseases may pave the way for the development of novel targeted therapies.

**Figure 3:**
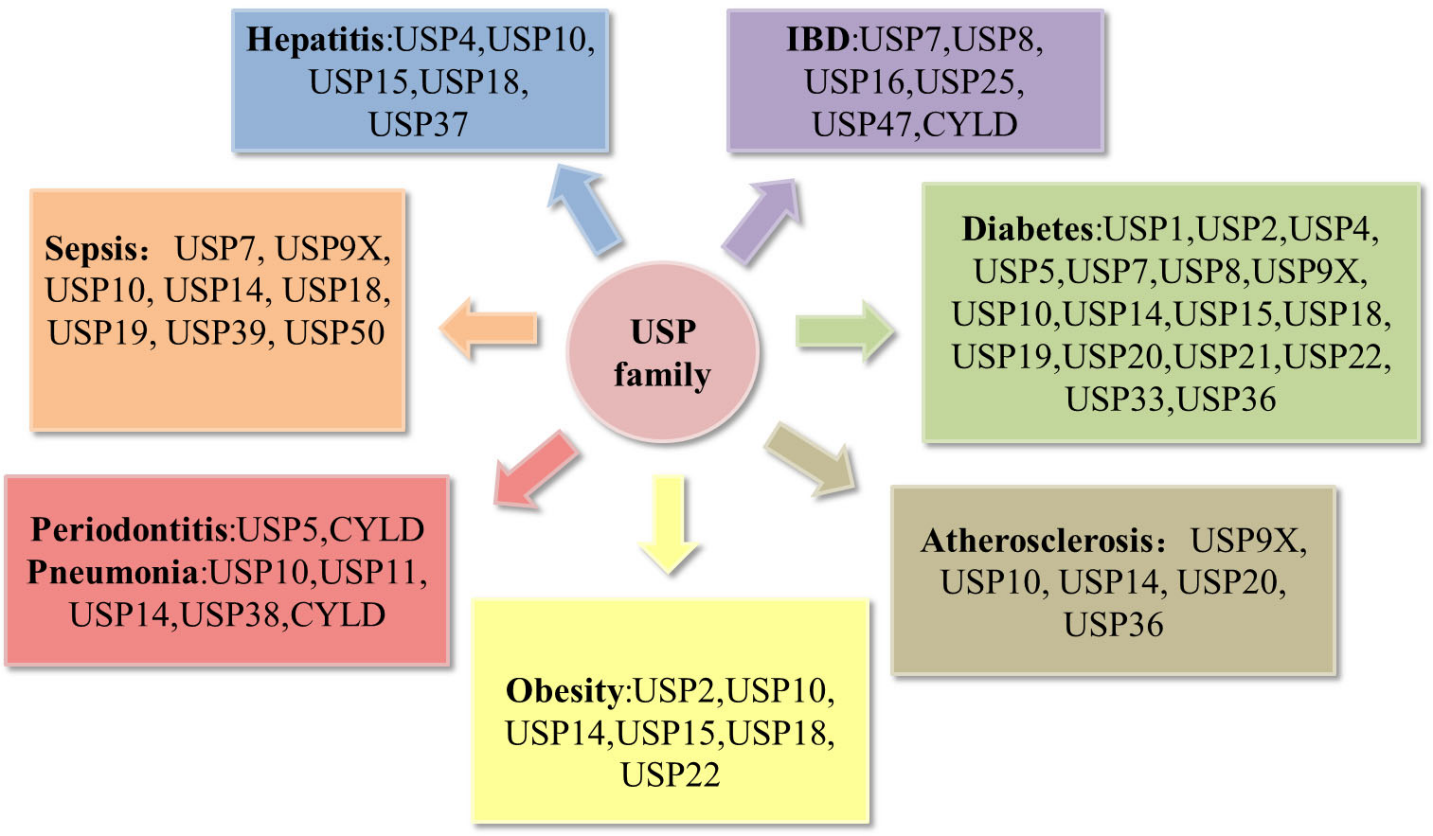
Deregulation of the USP family in inflammatory diseases. Pathological alterations of the USP family can affect the development of inflammatory diseases, including periodontitis, pneumonia, atherosclerosis, obesity, hepatitis, sepsis, inflammatory bowel disease (IBD), diabetes.

Recent reviews have notably underscored the correlation between USPs and metabolic disorders (16), as well as their involvement in bone-related inflammatory ailments such as osteoarthritis and rheumatoid arthritis (17). A multitude of studies have effectively elucidated the pivotal roles that USPs play in the landscape of inflammatory diseases. In this review, we choose the most prevailing and focal inflammatory diseases associated with USPs, including periodontitis, pneumonia, atherosclerosis, inflammatory bowel disease, sepsis, hepatitis, diabetes, and obesity (**Figure 3**). Moreover, we highlight a comprehensive overview of the pathogenesis of the USPs in these inflammatory diseases, emphasizing the involvement of post-translational modifications in inflammatory responses. Our review aims to pave the way for future prospect of targeted therapies in these inflammatory diseases.

## 2 Roles of USPs in inflammatory diseases

### 2.1 The regulation of USPs in the context of epigenetic regulatory network

Epigenetic mechanisms exert control over gene expression at both the transcriptional and post-transcriptional levels, including DNA methylation, RNA methylation, RNA interference, and histone modifications (18–20). Ubiquitin-mediated degradation and the reverse process of deubiquitination regulate histones as part of the epigenetic machinery. Epigenetic mechanisms play an active role in reshaping the cellular transcriptome, thereby governing gene expression, cell differentiation, and development (20).

Innate and adaptive immune reactions are related to epigenetic modification (21). Remarkably, epigenetic regulation is a crucial player in the onset and development of inflammatory response (22). Numerous studies have highlighted the roles of USPs in various inflammatory diseases by modifying epigenetics. USPs play crucial roles in epigenetic mechanisms by regulating certain post-translationally modified epigenetic factors, such as NF-κB and p53, thereby influencing inflammation (23, 24). For example, USP9X can interact with Bcl10 of the Carma1-Bcl10-Malt1 (CBM) complex and remove the TCR-induced ubiquitin chain from Bcl10, thus promoting NF-κB activation induced by TCR signaling pathway, and then actively affecting the cytokine production, T cell proliferation and differentiation (24). Additionally, USPs can interfere with the stability of proteins involved in epigenetic mechanisms, such as histone deacetylases, regulating the termination of DNA repair and the reorganization of chromatin structure (25). USP11 can interfere with the chromatin remodeling NuRD complex, and coordinate with NuRD-associated histone deacetylation to regulate the DNA repair process and genomic stability (25). Overall, USPs play crucial roles in regulating the inflammatory response by removing Ub from targeted protein complexes and affecting the stability of epigenetic factors, thereby regulating inflammation-related gene expression and the transmission of epigenetic information.

### 2.2 Roles of USPs in periodontitis

Periodontitis, a chronic inflammatory disease, is characterized by the inflammation of the pocket wall, the resorption of alveolar bone, the formation of periodontal pocket, and the separation between gingiva and tooth, as well as the loss of tooth (26). Moreover, periodontitis is the primary cause of tooth loss in adults, and it leads to periodontal tissue damage and is associated with a variety of systemic complications, such as diabetes, obesity, cardiovascular disease, and so on (27–29). Numerous studies have stated periodontitis is mainly caused by the imbalance of the oral microbiota and host resistance (30), associated with environment factors, host factors and genetic factors (31). As one of the epigenetic modifications, USPs are key regulators of the inflammation responses in periodontitis (32).

Recent studies have shed light on the contrasting roles of USP5 and CYLD in periodontitis, offering potential avenues for therapeutic interventions (**Table 1**). It has been reported that USP5 is upregulated in the gingival crevicular fluid and gingival tissues of patients with periodontitis, showing a positive correlation with proinflammatory factors through the STAT3 signaling pathway, which exacerbates the inflammatory response in chronic periodontitis (33). Furthermore, CYLD has been reported to ameliorate alveolar bone loss in mouse models of periodontitis by regulating the number and activity of osteoclasts, as well as the genes related to osteogenesis and osteoclastogenesis in alveolar bones (34, 35). CYLD can also modulate the NF-κB signaling pathway, which plays a crucial role in the inflammatory response and osteoclast mediation in periodontitis (34).

**Table 1:** USPs and periodontitis.

Enzymes	Function	Mechanism	References
USP5	Aggregating the inflammatory response of chronic periodontitis	Regulating STAT3 signaling	(33)
CYLD	Ameliorating alveolar bone loss	Regulating the osteoclast number and activity, as well as osteogenesis and osteoclastogenesis genes of alveolar bones; Regulating NF-κB signaling pathway	(34, 35)

### 2.3 Roles of USPs in pneumonia

Pneumonia is a prevalent respiratory system inflammatory disease, which can be caused by a variety of pathogens. The clinical symptoms include cough, phlegm production, chest pain, fever, as well as severe complications such as acute lung injury (ALI) and pulmonary fibrosis (36, 37). However, there is still a lack of accurate treatment to cure pneumonia, necessitating the exploration of better therapeutic targets. Recently, it has been reported the key roles of USPs in pneumonia, which may provide novel insights that could have clinical implications for the management of the disease (**Table 2**).

**Table 2:** USPs and pneumonia.

Enzymes	Function	Mechanism	References
USP10	Promoting mucociliary clearance and the removal of pathogens to cure pneumonia	Interacting with the bacterial toxin	(38)
USP11	Deteriorating pneumonia	Deubiquitinating and stabilizing LPA1, enhancing LPA1-mediated proinflammatory effects	(39)
USP14	Promoting pneumonia, increasing the apoptosis of lung epithelial cells	Regulating I-κB stability, increasing the expression of PARP-1 and pro-apoptotic proteins, depressing the expression of anti-apoptotic proteins, inhibiting the lung epithelial cell growth	(40–42)
USP38	Alleviating the pneumonia response and the bleomycin-induced pulmonary fibrosis	Deubiquitinating IL-33 receptor, negatively regulating the IL-33-triggered signaling pathway and autophagic degradation	(37)
CYLD	Exacerbating lung infections, ALI, bacterial translocation, and lethality, inhibiting injury-induced fibrotic responses	Inhibiting NF-κB signaling pathway, suppressing the expression of PAI-1, inhibiting TGF-β signaling pathway	(36, 43–47)

Several studies have reported that USP11 and USP14 contribute to the aggravation of inflammation in LPS-induced pneumonia (39–41). USP11 exacerbates pneumonia by deubiquitinating and stabilizing LPA1, a proinflammatory factor, enhancing LPA1-mediated proinflammatory effects (39). Knockdown or pharmaceutical inhibition of USP11 alleviates the lung damage induced by LPS in mice (39). Furthermore, USP14 plays a proinflammatory role in pneumonia (40, 41). The overexpression of USP14 in mouse models leads to the degradation of I-κB, increasing the release of factors such as TNF-α and IL-8 in lung epithelial cells (41). Besides, PARP-1 is an important binding enzyme of pneumonia signaling pathway (42). In human lung epithelial cells, it has been demonstrated that USP14 aggravates the inflammatory response by interacting with PARP-1 and increasing its expression (40). Furthermore, USP14 upregulates the expression of pro-apoptotic proteins while downregulating the anti-apoptotic protein (40). This leads to increased apoptosis of lung epithelial cells and inhibition of cell growth, which can contribute to severe complications in pneumonia (40).

In contrast, some USPs show the anti-inflammatory role in pneumonia. USP38 has been shown to alleviate the inflammatory response in lung and bleomycin-induced pulmonary fibrosis in mouse models (37). This effect is achieved through deubiquitinating the IL-33 receptor and negatively regulating the IL-33-triggered signaling pathway and autophagic degradation (37).

In the early stages of infection, Klebsiella can evade the host defense immunity system by causing a lack of inflammatory response (43). CYLD is known to suppress the NF-κB signaling pathway during the initial inflammatory response triggered by Escherichia coli, Klebsiella, and Streptococcus pneumoniae, which ultimately exacerbates subsequent lung infections (43–45). Notably, CYLD deficiency offers protection against Streptococcus pneumoniae pneumolysin (PLY)-induced ALI, bacterial translocation, and lethality (36). Furthermore, CYLD contributes to the worsening of ALI by suppressing the expression of plasminogen activator inhibitor 1 (PAI-1), a recognized biomarker of tissue injury that plays a crucial role in tissue repair (36, 46). However, it’s important to note that after infection with Streptococcus pneumoniae, CYLD serves as a critical negative regulator for injury-induced fibrotic response by inhibiting TGF-β signaling pathway (36, 47).

Pathogens can also impact host deubiquitinating enzymes to contribute to disease progression. For example, Cystic Fibrosis Transmembrane Conductance Regulator (CFTR) is a secretory chloride channel, which plays an important role in mucociliary clearance by human airway epithelial cells and the innate immune response in the lung (38). Pseudomonas aeruginosa secretes the bacterial toxin Cif, which inhibits USP10, leading to decrease USP10-mediated deubiquitination of CFTR and increase CFTR degradation in lysosomes, causing the weaker mucociliary clearance and the harder removal of pathogens to cure pneumonia (38).

Furthermore, there are some drug discoveries about USP and pneumonia, such as QingFeiPaiDu decoction and wogonoside. Mechanically, it decreases the expression of USP14 to reduce the phosphorylation of LPS-stimulated transcription factor 2(ATF2), an important regulator of cytokines, to play an anti-inflammatory role (48).

### 2.4 Roles of USPs in atherosclerosis

Atherosclerosis, an inflammatory disease primarily situated within arterial walls, arises due to the aberrant accumulation of low-density lipids (LDL) and lipid proteins (49–51). This process involves the participation of diverse cell types, including smooth muscle cells (SMCs), macrophages, neutrophil granulocytes, endothelial cells (ECs), and other leukocytes (49–51). Existing research has demonstrated the pivotal roles of NLRP3 inflammasome, IL-1β, and TNF in atherosclerosis (49, 51). Consequently, there is an imperative to identify more precise upstream or downstream targets to facilitate a more accurate therapeutic approach for atherosclerosis (49, 51).

Recent investigations have significantly highlighted the substantial involvement of USPs in atherosclerosis progression. Studies indicate that USP9X, USP10, USP14, USP17, USP20, and USP36 play regulatory roles in the atherosclerotic process (**Table 3**). Macrophages intake an excess of lipids, leading to the proliferation of macrophages and secrete inflammatory factors, which causes the formation of foam cells, thus aggravating atherosclerosis (49, 52, 54). This process hinges on receptor activity, including CD36, SR-A, and SR-B1 (57).

**Table 3:** USPs and atherosclerosis.

Enzymes	Function	Mechanism	References
USP9X	Inhibiting the intake of lipid of macrophages, the formation of foam cells and inflammatory response	Removing the polyubiquitin of SR-A63, regulating ECs	(52, 53)
USP14	Inhibiting inflammation in ECs; promoting the multiplication and mobilization of HASMCs; promoting the intake of ox-LDL; increasing the formation of foam cells	Inhibiting the activity of NF-κB and the degradation of its related regulation factors stimulated by ox-LDL, removing ub from CD36 to stabilize CD36 protein	(54, 55)
USP36	Promoting atherosclerosis; promoting the multiplication and mobilization of HASMCs	Regulating miR-182-5p signaling pathway	(55, 56)
USP10	Promoting the intake of oxidized LDL; increasing the formation of foam cells	Removing Ub from CD36 to stabilize CD36 protein	(57)
USP20	Decreasing atherosclerosis induced by TNF and IL-1β in SMCs	Deubiquitinating RIPK1, TRAF6	(58, 59)

Notably, USP9X has been identified as a suppressor of macrophage lipid intake, exhibiting lower expression levels in atherosclerosis compared to normal cells. Mechanically, USP9X removes polyubiquitin chains from SR-A63, thus inhibiting lipid intake by macrophages, impeding foam cell formation, and reducing the ensuing inflammatory response (52). Furthermore, USP9X, alongside USP14 and USP36, contributes to the regulation of ECs (53, 54, 60). Respectively, in atherosclerosis patients, USP14 is observed to be downregulated in ECs, and it inhibits the inflammatory response in ECs (54). This downregulation hampers the activity of NF-κB and the degradation of its associated regulatory factors, which are stimulated by oxidized LDL (ox-LDL) (54). On the other hand, USP36 contributes to atherosclerosis progression through the exosomal microRNA-197-3p signaling pathway (60). Furthermore, both USP14 and USP36 play roles in the proliferation and mobilization of human aortic smooth muscle cells (HASMCs) (55, 56). Additionally, USP14 and USP10 can increase the formation of foam cells via removing Ub from CD36, thereby stabilizing the CD36 protein and promoting the uptake of ox-LDL (57, 61). To briefly sum up, USP14 has shown effects on ECs, SMCs and foam cells in the development of atherosclerosis (54, 55, 61). Moreover, USP20 alleviates inflammation in SMCs in TNF and IL-1β-induced atherosclerosis by deubiquitinating RIPK1, a vital factor of inflammation and cell death (58, 59).

### 2.5 Roles of USPs in inflammatory bowel disease

Inflammatory bowel disease (IBD) is a chronic inflammatory disorder of the gastrointestinal tract, encompassing ulcerative colitis (UC) and Crohn’s disease (CD) (62). The etiology and mechanisms underlying IBD are complex, and extensive studies have implicated the involvement of USPs in the disease (**Table 4**).

**Table 4:** USPs and inflammatory bowel disease.

Enzymes	Function	Mechanism	References
USP7	Reducing the inflammation of IBD	Deubiquitinating and increasing the expression of Foxp3	(63, 64)
USP8	Alleviating IBD	Negatively modulating NOD2-induced cytokine secretion	(65)
USP16	Aggravating IBD	Upregulating inflammatory factors, promoting the proliferation and differentiation of T cells	(66, 67)
USP25	Promoting inflammation	Decreasing hyper-immune responses against bacterial infections	(68)
USP47	Alleviating IBD	Repressing NF-κB signaling pathway in intestinal epithelial cells	(69)
CYLD	Restricting the IBD inflammation	Inhibiting the excessive production of IL-18 through deubiquitinating NLRP6 in the colonic mucosa	(70, 71)

Recent studies have highlighted the significant impact of USPs in IBD. USP7, USP47, USP8, and CYLD have been implicated in alleviating IBD. Foxp3 is an essential factor for the development of regulatory T cells (Tregs) (63). USP7 increases the quantity and function of Tregs to maintain self-tolerance and reduce the inflammation of IBD by directly deubiquitinating and enhancing the expression of Foxp3 (63). Cambogin, a potential drug, can enhance the effect of USP7 and holds promise as a future treatment for IBD based on this mechanism (64). Additionally, USP47 is observed to be downregulated in chronic inflammatory mucosal tissue of CD and UC in patients with IBD (69). Knocking down USP47 in mice makes it easier to induce IBD and results in a more severe inflammatory response and tissue damage (69). This occurs by suppressing the NF-κB signaling pathway in intestinal epithelial cells (69). NOD2 is reported to be a strong genetic factor associated with IBD, and USP8 can negatively regulate NOD2-induced IL-8 and IL-6 in bone marrow-derived macrophages to inhibit inflammation in IBD (65). CYLD, another USP, restricts IBD inflammation in the colonic mucosa by inhibiting excessive production of IL-18 through deubiquitinating NLRP6 (70). However, CYLD is significantly downregulated in IBD patients (71). This downregulation leads to severe infection by adherent-invasive Escherichia coli (AIEC), activation of NF-κB, and degradation of IκB-α, thus exacerbating IBD (71).

However, USP16 and USP25 has the contrary impact on IBD. USP16 exhibits increased expression in patients with IBD and contributes to disease inflammation (66, 67). It has demonstrated that USP16 promotes the proliferation and differentiation of T cells, thereby increasing CD4^+^ T cell infiltration and aggravating IBD (66). Furthermore, USP25 significantly regulates proinflammatory cytokines in the colon (68). Knockdown of USP25 leads to hyper-immune responses against bacterial infections, thereby restricting bacterial replication and inflammation (68).

### 2.6 Roles of USPs in hepatitis

The liver, being the body’s primary detoxifying organ, is susceptible to hepatitis caused by various factors. Hepatitis can be classified into different types, such as viral hepatitis, autoimmune hepatitis, nonalcoholic steatohepatitis (NASH), and others (72, 73). Viral hepatitis has the largest population of hepatitis in the world, including HAV, HBV, HCV, HDV and HEV (73). The development of hepatitis involves complex mechanisms, often characterized by liver inflammation, damage, scarring, and the potential progression to hepatocirrhosis, liver cancer, and even death (73). Recent research has shed light on the involvement of several USPs in the development and progression of hepatitis. These USPs regulate various cellular processes that are crucial to the pathogenesis of hepatitis, including inflammation, immune response, viral replication, and cell death (**Table 5**). By modulating these processes, USPs contribute to the intricate mechanisms underlying hepatitis.

**Table 5:** USPs and hepatitis.

Enzymes	Function	Mechanism	References
USP4	Aggravating inflammation and fibrosis in the liver	Regulating TGF-β1 signaling pathway	(74)
USP10	Alleviating hepatic histological steatosis, inflammation, and fibrosis	Promoting autophagy	(75)
USP15	Influencing viral hepatitis, contributing to HCV transmission in hepatocytes	Upregulating the transactivation activity and stability of HBx, regulating viral RNA translation and lipid metabolism	(76–78)
USP18	Stimulating HCV and HBV replication, promoting viral entry and infectivity, promoting hepatic inflammatory responses	Inhibiting IFN-α and utilizing the IFN stimulated gene 15 (ISG15)/USP18 pathway, fostering a cellular environment characterized by CD81 upregulation, attenuating the effects of type I and type III IFNs, deubiquitinating TAK1	(79–92)
USP37	Influencing viral hepatitis	Reacting with the HBx	(78)

#### 2.6.1 Viral hepatitis

In viral hepatitis, various factors such as LPS, TNF-α, IL-17A, ISG15, Ach, and IFN-λ4 can increase the expression of USP18 in hepatocytes, leading to the inhibition of IFN signaling and attenuating the antiviral activity of IFN-α (79–83). The upregulation of USP18 in hepatocytes has been associated with poor outcomes in IFN-α therapy for chronic HBV and HCV patients (84–86). Conversely, silencing USP18 can enhance the effectiveness of IFN treatment by improving IFN-α2a signaling, inducing IFN-stimulated genes, and enhancing antiviral activity (84–86). Furthermore, USP18 promotes HCV and HBV replication by specifically inhibiting IFN-α and utilizing the IFN stimulated gene 15 (ISG15)/USP18 pathway, as well as fostering a cellular environment characterized by CD81 upregulation and promoting viral entry and infectivity (87, 88). Additionally, USP18 dampens the effects of type I and type III IFNs by exerting negative feedback control through its regulatory role (89). Moreover, USP18 also binds to and deubiquitinates TAK1, thereby promoting hepatic inflammatory responses (90–92).

In addition, USP15 and USP37 can react with the HBV X protein (HBx), which regulates viral replication in viral hepatitis (76–78). USP15 enhances the transactivation activity and stability of HBx, potentially influencing HCV transmission in hepatocytes by modulating viral RNA translation and lipid metabolism (76–78). Moreover, Qisheng Li et al. have employed functional genomics approaches and HCV model systems to demonstrate the involvement of USP11 in HCV-mediated translation, but the finer details of its regulatory mechanisms remain elusive (93).These findings highlight the involvement of specific USPs in viral hepatitis, particularly their impact on IFN signaling, viral replication, and hepatic inflammatory responses.

#### 2.6.2 Autoimmune hepatitis and nonalcoholic steatohepatitis

USP4 shows significant upregulation in autoimmune hepatitis (74). The intervention using Vialinin A and liver X receptor α (LXRα)-induced cannabinoid receptor 2 (CB2) has been found to reduce the level of USP4, leading to alleviate inflammation and fibrosis in the liver (74). Additionally, it has been reported that CB2 inhibited USP4, leading to the stabilization of TGF-β1R, and subsequently ameliorating hepatic autoimmune hepatitis (74). Furthermore, USP10 plays a role in attenuating hepatic steatosis in nonalcoholic steatohepatitis (NASH) through the promotion of autophagy, which alleviates hepatic steatosis, inflammation, and fibrosis (75).

### 2.7 Roles of USPs in sepsis

Sepsis is characterized by the deadly dysfunction of the host’s immune system in response to infections (94). The serious systemic inflammation derives from localized infections or sterile inflammatory diseases (95). Current approaches mainly involve supportive measures such as antibiotics and oxygen therapy, lacking precise targeted therapies (95). Researchers have underscored the complexity of small molecule mechanisms, including molecules like TNF-α, which are being explored as potential targeted therapy (95). However, the more precise targeted molecules are necessary to improve sepsis treatment.

Recently, there have been some studies on the regulation of USPs in the development of sepsis (**Table 6**). In sepsis, the level of USP9X is upregulated in CD8+ T cells, and this upregulation contributes to the development of sepsis by causing dysfunction in CD8+ T cells (96). This dysfunction can be reversed by WP1130, an inhibitor of USP9X (96). In sepsis, the transcription factor SRY-box 9 (SOX9) is highly expressed in cardiac muscle cells, contributing to cardiac damage (97). USP7 exacerbates the adverse effects of sepsis on the heart by deubiquitinating SOX9 and leading to an upregulation of its expression (97). Moreover, USP10 has been identified as a protective factor for renal tubular epithelial cells against acute kidney injury in LPS-induced sepsis (98).

**Table 6:** USPs and sepsis.

Enzymes	Function	Mechanism	References
USP9X	Promoting sepsis	Leading to the dysfunction of CD8 + T cells	(96)
USP7	Aggravating the detriment of sepsis	Deubiquitinating and upregulating SOX9	(97)
USP10	Alleviating sepsis	Protecting renal tubular epithelial cells from acute kidney injure induced by LPS	(98)
USP14	Promoting the release of cell factors induced by LPS	Interacting with TRAF6; deubiquitinating CBP and stabilizing it	(99)
USP18 and USP19	Alleviating sepsis and improving livability	Preventing the production of IL-6, IL-1β and TNF-α in sepsis, inhibiting the activation of NF-κB via deubiquitinating TAK1	(100, 101)
USP39	Decreasing the release of proinflammation factors and sepsis	Negatively modulating NF-κB signaling pathway; deubiquitinating and stabilizing IκBα	(102)
USP50	Aggravating LPS-induced sepsis	Stabilizing carnitine palmitoyltransferase 1a (CPT1a) and enhancing FAO	(103)

CBP, responsible for the release of cellular factors during sepsis, undergoes degradation due to ubiquitination (99). USP14 facilitates the release of cell factors in response to LPS during sepsis by deubiquitinating CBP and thereby stabilizing it (99). Recently, it has been proposed that a novel USP14 inhibitor, Neochromine S5, binds to USP14, diminishing its deubiquitination activity and disrupting the interaction between USP14 and TRAF6, which effectively reduces proinflammatory factors, and downregulates NF-κB and STAT1 signaling pathways (104). As a result, Neochromine S5 shows higher efficacy and safety in alleviating sepsis (104).

Moreover, both USP18 and USP19 have shown potential in mitigating sepsis and enhancing survivability (100, 101). They can curb the production of IL-6, IL-1β, and TNF-α in sepsis by inhibiting the activation of NF-κB through deubiquitination of TAK1 (100, 101). Furthermore, USP39 is downregulated in LPS-induced sepsis and it can decrease the release of proinflammation factors and subsequently sepsis-related inflammation (102). Mechanically, USP39 is recognized as a negative modulator of NF-κB signaling pathway through deubiquitinating and stabilizing IκBα (102). In sepsis, energy consumption changes from C_6_H_12_O_6_ to fatty acid oxidation (FAO) (103). USP50 plays a role in aggravating sepsis by stabilizing carnitine palmitoyltransferase 1a (CPT1a) and enhancing FAO (103).

### 2.8 Roles of USPs in diabetes

Diabetes is a chronic epidemic disease characterized by the presence of high levels of glucose in the blood, which can cause various complications, including diabetic retinopathy (DR), diabetic nephropathy (DN), diabetic neuropathic pain (DNP), diabetic foot (DF), diabetic cardiomyopathy (DCM), and so on (105–107). It is classified into type 1 diabetes (T1D) and type 2 diabetes (T2D). Recently, there have been numerous studies on USPs in diabetes, shedding light on the intricate roles of USPs in the disease. Further exploration of USPs in diabetes may provide insights into novel treatment strategies for the disease and its associated complications.

#### 2.8.1 Roles of USPs in type 1 diabetes

Type 1 diabetes (T1D) is an autoimmune disease characterized by the attack of T cells on pancreatic β cells, which are responsible for insulin production and the regulation of blood glucose levels (108). Several ubiquitin-specific proteases (USPs) have been implicated in the development of T1D (**Table 7**). DNA damage is highly expressed in diabetes and aggravates diabetes (109). Suppression of USP1 has been demonstrated to improve diabetes outcomes by the inhibition of DNA damage, preventing pancreatic β cell apoptosis, preserving insulin secretion, and enhancing β-cell maturation in human islets (109). In addition, the CLEC16a-NRDP1-USP8 complex mediates ubiquitin-dependent signaling that promotes mitophagy and maintains mitochondrial quality in β cells (110). This process contributes to the preservation of precise β-cell function and helps regulate blood glucose levels (110). McL-1 is one of the anti-apoptotic Bcl-2 protein family, which is reduced in islets in T1D patients, and USP9X modulates McL-1 protein turnover mediated by cytokines to prevent islet cell death in β cells (111). Moreover, the dysregulation of secretagogue-dependent USP9X deubiquitinase activity can lead to decreased insulin utilization (112).

**Table 7:** USPs and type 1 diabetes.

Enzymes	Function	Mechanism	References
USP1	Promoting the pancreatic β cell apoptosis, deteriorating diabetes	Promoting the DNA damage	(109)
USP8	Protecting β-cell function and regulating blood glucose levels	Promoting mitophagy and maintaining mitochondrial quality in β cells	(110)
USP9X	Reducing islet cell death, decreasing insulin utilization	Modulating McL-1 protein turnover mediated by cytokines	(111, 112)
USP18	Alleviating the inflammation and death of β-cell, attenuating the proinflammatory response of β-cells	Regulating IFN signaling, STAT signaling, the mitochondrial pathway of cell death, regulating the activation of three BH3 proteins, reducing the expression of MDA5 and double-stranded chemokine production induced by RNA, regulating the expansion of autoreactive CD8^+^ T cells	(113–115)

USP18-driven dendritic cells play a significant role in breaking immune tolerance in autoimmune diabetes (113). The genetic deletion of USP18 can mitigate the expansion of autoreactive CD8^+^ T cells and provide protection against autoimmune diabetes (113). In pancreatic β cells, USP18 acts as a crucial modulator of the IFN signaling pathway and three BH3 proteins, which exerts a significant impact on the inflammation and death of β cells (114). Suppression of USP18 promotes inflammation by STAT signaling and aggravates β-cell apoptosis induced by IFN through the cell death in mitochondria (114). Moreover, USP18 reduces the expression of MDA5, the T1D candidate gene, leading to the downregulation of double-stranded chemokine production induced by RNA and attenuate the proinflammatory response of β cells (115).

#### 2.8.2 Roles of USPs in type 2 diabetes

Type 2 diabetes (T2D) is characterized by chronic inflammation, immune factor activation, and impaired insulin secretion and sensitivity, leading to elevated blood glucose levels (116). Numerous studies have identified several USPs that play roles in T2D (**Table 8**). A study indicates that USP2A and USP2 can alter insulin sensitivity, and USP2A blocks obesity-induced insulin resistance through adipocyte-dependent mechanisms (117). Additionally, USP4 decreases the ubiquitination and degradation of insulin receptors, consequently leading to reduced insulin resistance (118). Gastrodin, a compound derived from Gastrodia elata, has been reported to enhance the expression of USP4 and may represent a novel targeted treatment for T2D (118).

**Table 8:** USPs and type 2 diabetes.

Enzymes	Function	Mechanism	References
USP2	Altering insulin sensitivity	Depending on adipocyte	(117)
USP4	Reducing insulin resistance	Decreasing the ubiquitination and degradation of insulin receptors	(118)
USP7	Resulting in delayed insulin negative feedback loop and sustained insulin signaling	Participating in the insulin signaling pathway by binding to PiT1 and deubiquitinating the insulin receptor substrate	(119)
USP14	Promoting hyperglycemia and glucose intolerance	Increasing the damage to ER	(120)
USP19	Enhancing obesity and glucose intolerance induced by high-fat diet	Regulating adipogenesis	(121)
USP20	Promoting weight gain induced by diet, upregulating the levels of serum and liver lipid, reducing insulin sensitivity and energy expenditure	Stabilizing HMGCR during the feeding state	(122)
USP21	Promoting obesity and T2D	Inhibiting an oxidized fiber phenotype in skeletal muscle	(123)
USP22	Inhibiting ferroptosis induced by HG	Stabilizing SIRT1	(124)
USP33	Promoting insulin sensitivity	Mediating ADRB2 deubiquitination	(125)

However, certain USPs can aggravate T2D. It has been reported that USP7 can delay the insulin negative feedback loop, leading to sustained insulin signaling by binding to PiT1 and deubiquitinating the insulin receptor substrate (119). In addition, endoplasmic reticulum (ER) stress plays a significant role in T2D, and sustained ER stress upregulates USP14 (120). The knockdown of USP14 reduces the damage to ER associated with glucose metabolism and improves hyperglycemia and glucose intolerance in obese mice (120). Moreover, inhibition of USP19 has shown promise in regulating adipogenesis and improving glucose intolerance and obesity induced by a high-fat diet (121). Subsequently, the upregulation of postprandial glucose and insulin levels promotes the phosphorylation of USP20 (122). Suppression of USP20 significantly reduces weight gain, lipid levels in serum and liver, and improves insulin sensitivity and energy expenditure (122). This is achieved by stabilizing HMG-CoA reductase (HMGCR), which serves as the rate-limiting enzyme in the cholesterol biosynthesis pathway (122). In addition, ablation of USP21 in skeletal muscle enhances energy expenditure by stimulating an oxidative fiber phenotype that inhibits obesity and T2D (123). In T2DM mice models, ferroptosis is observed in pancreatic β cells (124). USP22 stabilizes Sirt1 to inhibit ferroptosis induced by HG (124). Furthermore, USP33 participates in the deubiquitination of β2-adrenergic receptor (ADRB2), stimulating insulin sensitivity in skeletal muscle (125).

#### 2.8.3 Roles of USPs in diabetic retinopathy

Diabetic retinopathy (DR) is a common microvascular complication of diabetes, and endothelial barrier integrity is important for vascular steady (126). Several pharmacological inhibitors of USP1 have been identified as potential treatments for DR, such as Primaquine Diphosphate, as they can alleviate β-cell death or inhibit vascular endothelial growth factor (VEGF)-induced leakage, thus improving DR outcomes (109, 127, 128). In DR patients, USP14 can modulate inflammatory response through the TGF-β1 signal transduction, IκBα and NF-κB signaling pathway s in HG-treated Müller cells, as well as reactive oxygen species (ROS) (126).This modulation plays a role in mediating the progression of DR (126) (**Table 9**).

**Table 9:** USPs and diabetic retinopathy, diabetic nephropathy, diabetic neuropathic pain, diabetic foot, diabetic cardiomyopathy.

USPs and Diabetic Retinopathy			
Enzymes	Function	Mechanism	References
USP1	Improving DR outcomes	Alleviating β-cell death, inhibiting VEGF-induced leakage	(109, 127, 128)
USP14	Leading to severer DR	Activating inflammatory response through the TGF-β1 signal transduction, IκBα and NF-κB signaling pathway s in HG-treated Müller cells, as well as reactive oxygen species (ROS)	(126)
USPs and Diabetic Nephropathy			
Enzymes	Function	Mechanism	References
USP9X	Protecting renal epithelial cells and alleviating DN	Preventing EMT induced by HG in NRK-52E cells, downregulating TGF-β1 and FN	(129, 130)
USP14	Aggravating podocyte injury and DN	Interacting with SPAG5-AS1	(131)
USP15	Increasing HG-induced podocyte apoptosis, oxidative stress and inflammation	Reducing the expression of Nrf2 target genes and Nrf2 activation in podocytes	(132)
USP22	Alleviating diabetic renal fibrosis, aggravating HG-induced apoptosis and inflammation in podocytes	Activating Sirt1 signaling pathway	(133–135)
USP36	Aggravating DN	Deubiquitinating DOCK4 and aggravating GEF-mediated EMT	(136)
USPs and Diabetic Neuropathic Pain			
Enzymes	Function	Mechanism	References
USP5	Affecting the DNP	Interacting with Cav3.2	(137, 138)
USP15	Alleviating DNP	Promoting the ubiquitination and degradation of Nrf2, reducing the expression of G6PD	(106, 137)
USPs and Diabetic Foot			
Enzymes	Function	Mechanism	References
USP7	Slowing the progression of diabetic wound healing	Upregulating in HUVECs and diabetic foot ulcers	(107)
USP30	Inhibiting wound healing in diabetic rats	Activating NLRP3 inflammasomes by deubiquitinating NLRP3	(105)
USPs and Diabetic Cardiomyopathy			
Enzymes	Function	Mechanism	References
USP8	Reducing blood glucose	Interacting with Parkin	(104)
USP10	Reducing the risk of MI	Deubiquitinating NICD1, modulating Notch signaling pathway	(139)

#### 2.8.4 Roles of USPs in diabetic nephropathy

Epithelial-mesenchymal transition (EMT) has a remarkable influence on diabetic nephropathy (DN) (129). In the kidney tissues of db/db mice and HG-induced NRK-52E cells, the expression of USP9X protein was dramatically decreased (129). USP9X has been identified as a key regulator in preventing HG-induced EMT and protecting renal epithelial cells in DN, while knockdown of USP9X aggravates the EMT process in HG-induced NRK-52E cell (129). Moreover, USP9X alleviates the development of diabetic renal fibrosis by downregulating TGF-β1 and fibronectin (FN), two markers of fibrosis in glomerular mesangial cells (GMCs) (130).

In contrast, it has been indicated that some USPs may exacerbate DN. The development of podocytes serves as a clinical marker for DN (131). Dysfunctional autophagy plays a significant role in podocyte injury (131). In human podocytes, USP14 can negatively regulate autophagy and aggravate podocyte injury by deubiquitinating and stabilizing SPAG5, which inhibits autophagy (131). Furthermore, USP15 is increased in podocytes upon HG stimulation, and the inhibition of USP15 reduces HG-induced podocyte apoptosis, oxidative stress and inflammation by enhancing the expression of Nrf2 target genes and Nrf2 activation (132). HeQing Huang et al. have reported that USP22 alleviates diabetic renal fibrosis, while Qin Huang et al. have indicated that USP22 aggravates HG-induced apoptosis and inflammation in podocytes (133, 134). Therefore, USP22 may affect the progression of DN from different aspects with different environments, more researches are needed to reveal the roles of USP22 in the progression of DN. Teneligliptin, a drug for T2D treatment, can stabilize USP22 to delay the progression of DN through activating Sirt1 signaling pathway (135). Besides, the expression of USP36 is increased in diabetic renal tubular epithelial cells, which has a great relationship with upregulated EMT (136). USP36 contributes to DN progression by directly deubiquitinating cytokinin 4 (DOCK4) and aggravating guanine nucleotide exchange factor (GEF)-mediated EMT (136) (**Table 9**).

#### 2.8.5 Roles of USPs in diabetic neuropathic pain

Cav3.2 calcium channels are proteins involved in nociceptive transmission, which are increased in response to nerve damage and peripheral neuroinflammation (137). Disrupting the interaction between Cav3.2 and USP5 using the TAT-cUBP1-USP5 peptide has been shown to reduce the levels of the Cav3.2 calcium channel *in vitro*, which attenuates thermal hyperalgesia in diabetic neuropathy animals (137, 138). Furthermore, USP15 has been found to enhance the current of Cav3.2 in afferent neurons by mediating the deubiquitination of the channel (137). Additionally, USP15 promotes the degradation of Nrf2 and upregulates the expression of glucose-6-phosphate dehydrogenase (G6PD) (137). In contrast, Mir-497 has been shown to downregulate the expression of USP15, thereby alleviating diabetic neuropathic pain (106) (**Table 9**).

#### 2.8.6 Roles of USPs in diabetic foot

The expression of USP7 has been reported to be upregulated in human umbilical vein endothelial cells and in diabetic foot ulcers (107). Small molecule inhibitors of USP7, such as Quiazolin-4-one scaffold and HBX 41108, have demonstrated the ability to accelerate wound healing (107). Furthermore, USP30 has been identified as a regulator of NLRP3 inflammasomes by deubiquitinating NLRP3 and activating its function (105). In diabetic rats, Mf-094, an inhibitor of USP30, can reduce NLRP3 expression and its downstream target caspase-1 p20, therefore alleviating the inflammation and promoting wound healing (105) (**Table 9**).

#### 2.8.7 Roles of USPs in diabetic cardiomyopathy

In diabetic conditions, excessive production of reactive oxygen species (ROS) can cause mitochondrial damage, leading to myocardial damage (104). Parkin facilitates clearance of damaged mitochondria (104). Studies have shown that in diabetic heart tissue, USP8 promotes mitophagy by deubiquitinating Parkin and facilitating the recruitment of Parkin to mitochondria, thereby protecting from myocardial injury (104). Patients with T2D are at increased risk for myocardial infarction (MI) (139). USP10, a deubiquitinating enzyme, modulates the Notch signaling pathway by targeting NICD1, the receptor of Notch1 (139). This pathway plays a vital role in the regulation of myocardial fibrosis (139). Follistatin-like protein 1 (FSTL1) has been found to protect cardiac fibroblasts from injury induced by diabetes mellitus-associated myocardial infarction (DM-MI). FSTL1 exerts its protective effects by downregulating fibrosis markers and upregulating USP10/Notch1 signaling, thereby inhibiting myocardial fibrosis and apoptosis (139) (**Table 9**).

### 2.9 Roles of USPs in obesity

Obesity, a significant health issue of the 21st century, is associated with the activation of the innate immune system, expansion of adipose tissue, and various metabolic disturbances (140). It gives rise to numerous long-term complications, including diabetes, cardiovascular disease, and non-alcoholic fatty liver disease (NAFLD) (141). There are several causes of obesity, such as food intake, adipogenesis and the activation of inflammation (142). In recent years, there has been a growing interest in the roles of USPs in research related to obesity (**Table 10**). Several USPs, including USP2, USP10, USP14, USP15, USP18, and USP22, have been associated with obesity and related metabolic disorders.

**Table 10:** USPs and obesity.

Enzymes	Function	Mechanism	References
USP2	Increasing the production of hepatic glucose and promoting glucose intolerance	Inducing HSD1 and glucocorticoid signaling pathway in liver	(143, 144)
USP10	Inhibiting glucose production and adipogenesis in hepatocytes	Removing AMPK inhibitory ubiquitin residues to regulate AMPK phosphorylation and dephosphorylation, inhibiting the ubiquitination and degradation of Sirt6	(145–149)
USP14	Promoting hepatic triglyceride accumulation, regulating human enterocyte differentiation, and influencing obesity	Increasing the stability of FASN	(150)
USP15	Affecting adipocyte differentiation and fat droplet formation	Related to FABP4	(151, 152)
USP18	Improving lipid metabolism and insulin sensitivity, improving insulin sensitivity	Deubiquitinating TAK1, inhibiting TAK1 phosphorylation, inhibiting NF-κB signaling pathway, upregulating FTO	(92, 153, 154)
USP22	Reducing hepatic steatosis and obesity	Stabilizing Sirt1 protein, regulating Sirt1-dependent mitochondrial respiration	(145)

In diet-induced obese mice, it is reported that USP2 can increase the production of hepatic glucose and promote glucose intolerance (143). USP2 is regulated by peroxisome proliferator-activated receptor γ coactivator-1α (PGC-1α) and influences hepatic glucose metabolism by inducing 11β-hydroxysteroid dehydrogenase 1 (HSD1) and glucocorticoid signaling pathways (143). Furthermore, 3,3’-diindolylmethane, derived from cruciferous vegetables, inhibits adipogenesis in preadipocytes by targeting USP2 activity, thereby inhibiting high-fat diet-induced obesity (144).

NAFLD is caused by mitochondrial dysfunction with persistent imbalance between energy intake and expenditure, and USP10 is a negative regulator of NAFLD (145). However, the protein level of USP10 in liver is decreased in obese and NAFLD patients (146). USP10 inhibits glucose production and adipogenesis in hepatocytes, and its activity modulated by long noncoding RNA myocardial infarct-related transcript 2 (Mirt2) (147). Besides, USP10 improves metabolic dysfunction associated with obesity by regulating liver steatosis, inflammation, and insulin resistance (146, 148, 149). Mechanically, USP10 regulates AMPK phosphorylation and dephosphorylation by removing inhibitory ubiquitin residues (146, 148, 149). Additionally, it inhibits the ubiquitination and degradation of Sirt6 (146, 148, 149).

USP14 can promote hepatic triglyceride accumulation by increasing the stability of fatty acid synthase (FASN), which is the crucial enzyme for hepatic adipogenesis (150). Besides, human intestinal cell differentiation is altered during bariatric surgery, and USP14 can regulate human enterocyte differentiation and influence obesity (155).

USP15 plays a significant role in adipocyte differentiation and the formation of fat droplets. It has been reported that USP15 is closely and functionally related to fatty acid binding protein 4 (FABP4), which is abundant in mature adipocytes (151, 152).

USP18 is downregulated in obese mice, which can improve lipid metabolism and insulin sensitivity (92). In principle, TAK1-dependent signaling is vital for the development of hepatic insulin resistance (153). USP18 can improve insulin sensitivity by deubiquitinating TAK1 and inhibiting TAK1 phosphorylation to downregulate NF-κB signaling pathway and upregulate fat mass and obesity-related protein (FTO) (153, 154).

Furthermore, USP22 reduces hepatic steatosis and obesity by stabilizing Sirt1 protein and regulating Sirt1-dependent mitochondrial respiration (145).

## 3 Discussion

In recent years, extensive research has shed light on the mechanisms of ubiquitin-specific proteases (USPs) in inflammatory diseases. This review summarizes the important roles of the USPs in the onset and progression of inflammatory diseases, including periodontitis, pneumonia, atherosclerosis, inflammatory bowel disease, sepsis, hepatitis, diabetes, and obesity (40, 75). However, the functions of the USP family have not been fully elucidated (15).

While some USPs have similar functions in inflammation, their underlying mechanisms can differ. The inflammatory response is complex, and the interactions between USPs and inflammatory diseases vary depending on their substrates, tissue types, and environmental conditions (156). USPs participate in various signaling pathways due to the multitude of their substrates (157). Understanding how USPs select their substrates and express themselves specifically in targeted tissues may enable the development of accurate therapies for specific inflammatory diseases. Thus, exploring the role of USPs in inflammatory diseases represents a vast domain for further investigation (158).

Inflammatory diseases exhibit varying expressions of USPs in different cells and tissues. Most studies focus on immune cells or corresponding organ tissues, with only a few examining the expression of USPs in peripheral blood cells (159, 160). Therefore, further research is needed to investigate the role of USPs in other cells involved in inflammatory diseases, which may provide insights into disease phenotypes based on USP expression patterns.

USPs influence protein stability and can be regulated by small molecule inhibitors. Efforts have been made to develop drugs that target USPs for clinical applications (161, 162). For example, QingFeiPaiDu decoction and wogonoside have been reported to reduce USP14 levels, thereby alleviating pneumonia (48). These compounds are shown to reduce the phosphorylation of ATF2 and the NF-κB signaling pathway stimulated by LPS (48). However, due to the complex composition of these compounds, investigating the mechanisms presents a significant challenge (48). Furthermore, Gastrodin has been shown to upregulate the expression of USP4 and enhance the interaction between USP4 and insulin receptors to treat diabetes (118). Gastrodin has also been reported to increase the activity of pancreatic β-cells and stimulate insulin secretion, ultimately improving diabetes (118).

Some USP inhibitors have shown better pharmacological efficacy than previously known drugs and may overcome drug resistance through combination therapy. For example, it has been reported that administering USP7 alone or in combination with synergistic pathways significantly enhances DNA damage effects and overcomes treatment resistance (163).

Nonetheless, due to the high homology among members of the USP family, achieving specificity with USP inhibitors poses a significant challenge (164). This results in the existing inhibitors lacking specificity and often failing to exclusively target a single member of USPs, thereby somewhat limiting their utility. For instance, drugs like PR619 target a range of enzymes including USP2, USP4, USP5, USP7, USP8, USP15, USP20, USP28, USP47, UCHL1, UCHL3, UCHL5, highlighting the need to craft more selective agents (164). A prime example is the subsequent USP1 inhibitor ML323, exhibiting higher specificity compared to previous compounds like Pimozide and GW7647, thereby widening its scope of application (164). To achieve enhanced specificity in inhibitors, careful consideration of the chemical structure of USPs is needed (164). Nowadays, it is crucial to search for and apply suitable biological testing methods to screen and identify small molecule inhibitors of USPs. Currently, the existing methods include high-throughput screening (HTS), bioinformatics approaches, virtual screening and so on (165).

Furthermore, the diversity of pathways and physiological activities regulated by USPs has led to potential toxicity concerns (164). Deubiquitination virtually controls numerous aspects of human cellular biology and physiology, and any defects in these processes may lead to diseases (164). For instance, VLX1570 is an inhibitor of USP14. The clinical trial of VLX1570 in combination with dexamethasone for multiple myeloma patients was halted due to pulmonary toxicity (164). Besides, USP30 controls the import of mitochondrial proteins, suggesting potential toxic effects of the inhibitor of USP30 (166). Although numerous reagents have displayed promise *in vitro* and animal experiments, clinical trials validating their specific effects and side effects are scarce. It is vital to bridge the gap between experimental findings and clinical relevance through further investigations, including clinical trials, to establish the significance of USPs in diverse inflammatory conditions. Additionally, the solubility and stability of inhibitors in aqueous solutions significantly impact their pharmaceutical potential in clinical applications (164). These underscore the need for extensive exploration.

In summary, as we discover more phenotypes and mechanisms, USPs are increasingly recognized as essential modulators of inflammatory diseases. Further research will identify novel directions and effective approaches to improve therapeutic treatments. It is important to continue investigating the role of USPs in inflammatory diseases to enhance our understanding and develop targeted therapies for improved patient outcomes.

## Author contributions

RC: Writing – original draft. HZ: Writing – review & editing. LL: Writing – review & editing. JL: Writing – review & editing. JX: Writing – review & editing. JW: Writing – review & editing. HT: Writing – review & editing. YL: Writing – review & editing. TG: Funding acquisition, Writing – review & editing. MW: Conceptualization, Funding acquisition, Writing – review & editing.

## Glossary

**Table:**

USPs	Ubiquitin-specific proteases
DUBs	Deubiquitinating enzymes
Ub	Ubiquitin
UCHs	Ubiquitin carboxyl-terminal hydrolases
MJDs	Machado-Josephin disease proteins
OTUs	Ovarian tumor proteases
JAMMs	Josephins and JAB1/MPN/Mov34 metalloenzymes
STAT	Signal transducer and activator of transcription
ALI	Acute lung injury
PAI-1	Plasminogen activator inhibitor 1
TAK1	Transforming growth factor-β-activated kinase 1
TLR2	Toll-like receptor 2
CFTR	the Cystic Fibrosis transmembrane conductance regulator
ATF2	LPS-stimulated transcription factor 2
H. pylori	Helicobacter pylori
IBD	Inflammatory bowel disease
CD	Crohn’s disease
UC	Ulcerative colitis
TCR	T-cell antigen receptor
AIEC	Adherent-invasive Escherichia coli
ISG15	Interferon stimulated gene 15
HBx	HBV X protein
LXRα	Liver X receptor α
CB2	Cannabinoid receptor 2
NASH	nonalcoholic steatohepatitis
T1D	Type I Diabetes
T2D	Type 2 Diabetes
DN	Diabetic nephropathy
DR	Diabetic retinopathy
DNP	Diabetic neuropathic pain
G6PD	Glucose-6-phosphate dehydrogenase
DF	Diabetic foot
DCM	Diabetic cardiomyopathy
VEGF	Vascular endothelial growth factor
HUVECs	Human umbilical vein endothelial cells
HG	High glucose
ROS	Reactive oxygen species
EMT	Epithelial-mesenchymal transition
Nrf2	Nuclear factor-E2-related factor
ARE	Antioxidant response element
MI	Myocardial infarction
FSTL1	Follistatin like protein 1
ER	Endoplasmic reticulum
CREB	3', 5'-cyclic monophosphate response element binding
CBP	3', 5'-cyclic monophosphate response element binding protein
TβR1	Transforming growth factor-β1 receptor
SPAG5-AS1	Sperm-related antigen 5 antisense RNA1
HMGCR	HMG-CoA reductase
FN	Fibronectin
GMCs	Glomerular mesangial cells
ADRB2	β2-adrenergic receptor
DOCK4	Cytokinin 4
GEF	Guanine nucleotide exchange factor
NAFLD	Non-alcoholic fatty liver disease
PGC-1α	Peroxisome proliferator-activated receptor γ coactivator-1α
HSD1	11β-hydroxy steroid dehydrogenase 1
PiT1	Phosphate inorganic transporter 1
Mirt2	Myocardial infarct-related transcript 2
FASN	Fatty acid synthase
FABP4	Fatty acid binding protein 4
FTO	Obesity-related protein
