# Corrigendum: Roles of ubiquitin-specific proteases in inflammatory diseases

**DOI:** 10.3389/fimmu.2024.1392734

**Published:** 2024-03-06

**Authors:** Rui Chen, Hui Zhang, Linke Li, Jinsheng Li, Jiang Xie, Jie Weng, Huan Tan, Yanjun Liu, Tailin Guo, Mengyuan Wang

**Affiliations:** ^1^Center of Obesity and Metabolic Diseases, Department of General Surgery, The Third People’s Hospital of Chengdu, Affiliated Hospital of Southwest Jiaotong University, Chengdu, China; ^2^Department of Stomatology, The Third People’s Hospital of Chengdu, The Affiliated Hospital of Southwest Jiaotong University, Chengdu, Sichuan, China; ^3^College of Medicine, Southwest Jiaotong University, Chengdu, Sichuan, China; ^4^College of Materials Science and Engineering, Southwest Jiaotong University, Chengdu, Sichuan, China; ^5^Department of Pediatrics, Chengdu Third People’s Hospital, Chengdu, Sichuan, China

**Keywords:** deubiquitination, ubiquitin-specific proteases, inflammatory diseases, protein stability, targeted therapies

In the published article, there was an error regarding the affiliation(s) for Huan Tan. As well as having affiliation 1, they should also have affiliation 3 “College of Medicine, Southwest Jiaotong University, Chengdu, Sichuan, China”.

In the published article, there was an error regarding the affiliation(s) for Yanjun Liu. As well as having affiliation 3, they should also have affiliation 1 “Center of Obesity and Metabolic Diseases, Department of General Surgery, The Third People’s Hospital of Chengdu, Affiliated Hospital of Southwest Jiaotong University, Chengdu, China”.

In the published article, there was an error regarding the affiliation(s) for Tailin Guo. As well as having affiliation(s) 1, they should also have affiliation 3 “College of Medicine, Southwest Jiaotong University, Chengdu, Sichuan, China”.

The authors apologize for this error and state that this does not change the scientific conclusions of the article in any way. The original article has been updated.

